# Hepatectomy Based on Future Liver Remnant Plasma Clearance Rate of Indocyanine Green

**DOI:** 10.1155/2016/7637838

**Published:** 2016-06-23

**Authors:** Yuichiro Uchida, Hiroaki Furuyama, Daiki Yasukawa, Hiroto Nishino, Yasuhisa Ando, Toshiyuki Hata, Takafumi Machimoto, Tsunehiro Yoshimura

**Affiliations:** ^1^Department of Gastrointestinal and General Surgery, Tenri Yorozu Hospital, 200 Mishima-cho, Tenri, Nara 632-8552, Japan; ^2^Division of Hepato-Biliary-Pancreatic and Transplant Surgery, Graduate School of Medicine, Kyoto University, 54 Shogoin-kawahara-cho, Kyoto 606-8507, Japan

## Abstract

*Background*. Hepatectomy, an important treatment modality for liver malignancies, has high perioperative morbidity and mortality rates. Safe, comprehensive criteria for selecting patients for hepatectomy are needed. Since June 2011, we have used a cut-off value of ≧ 0.05 for future liver remnant plasma clearance rate of indocyanine green as a criterion for hepatectomy. The aim of this study was to verify the validity of this criterion.* Methods*. From June 2011 to December 2015, 212 hepatectomies were performed in Tenri Yorozu Hospital. Of these 212 patients, 107 who underwent preoperative computed tomography imaging volumetry, indocyanine green clearance test, and hepatectomy (excluding partial resection or enucleation) were retrospectively analyzed.* Results*. There was no postoperative mortality. Posthepatectomy liver failure occurred in 59 patients (55.1%) (International Study Group of Liver Surgery Grade A: 43 cases (40.2%), Grade B: 16 cases (15.0%), and Grade C: no cases). Operative morbidity greater than Clavien-Dindo Grade 3 occurred in 23 patients (21.5%). A low future liver remnant plasma clearance rate of indocyanine green was a good predictor for Grade B cases (area under curve = 0.804; 95% confidence interval, 0.712–0.895).* Conclusion*. Liver remnant plasma clearance rate of indocyanine green is a valid criterion for hepatectomy.

## 1. Introduction

Hepatectomy is an important treatment modality for liver malignancies. On the other hand, postoperative morbidity and mortality rates are still high. Posthepatectomy liver failure (PHLF), one of the most critical forms of morbidity, is closely correlated with postoperative mortality.

In 1993, Makuuchi's criteria [1] were proposed for hepatectomy in patients with underlying liver diseases. These criteria are based on presence or absence of ascites, preoperative total bilirubin concentration, and indocyanine green (ICG) retention rate at 15 minutes and have since been widely accepted in Japan. Makuuchi's criteria are probably appropriate for patients with basically healthy liver too and are used by many surgeons; however, patients who are ineligible for hepatectomy according to Makuuchi's criteria are frequently encountered. The safety of hepatectomy in such patients is still controversial. In 1980, Takasaki et al. reported that future liver remnant plasma clearance rate of ICG (rICGK) was useful for predicting posthepatectomy liver function [2]. It is easily calculated as follows: preoperative ICGK ×  % future remnant liver volume (RLV) and is also widely used in Japan. Nagino et al. and Yokoyama et al. reported that rICGK less than 0.05 is associated with a high incidence of perioperative mortality after hepatectomy for biliary cancer [3, 4], but the significance of rICGK on hepatectomy for other diseases has not been fully evaluated.

## 2. Methods

### 2.1. Patients

From June 2011 to December 2015, 212 patients underwent hepatectomy in Tenri Yorozu Hospital. Eighty-nine patients who had undergone limited resection (partial resection or enucleation) and 16 who had undergone different types of hepatectomy than had been planned preoperatively by computed tomography (CT) imaging were excluded because lack of data on future RLV prevented assessment of the validity of the rICGK ≧ 0.05 criterion in these patients. Thus, 107 patients who had undergone hepatectomy based on preoperative planning by computed tomography (CT) imaging volumetry were included in this study.

Patient characteristics, indications for hepatectomy, and types of hepatectomy are shown in Tables 1 and 2.

### 2.2. Resection Criteria

Only patients whose rICGK ≧ 0.05 were considered eligible for hepatectomy. Preoperative CT imaging volumetry was performed using SYNAPSE VINCENT version 2.0 (FUJIFILM, Tokyo, Japan). Preoperative portal embolism was performed in eight patients whose rICGK was less than 0.05. After this procedure, the rICGK became greater than 0.05 in all eight of these patients and all of them subsequently underwent hepatectomy. We have included these eight patients in this study.

### 2.3. Clinical Data Assessed

The clinical data we assessed included the following: age, sex, ICGK, ICG retention rate at 15 minutes (ICGR15), total liver volume, % RLV (future remnant liver volume/total liver volume − tumor volume × 100), rICGK, serum total bilirubin (T-Bil), serum albumin (Alb), platelet count (Plt), international normalized ratio of prothrombin time (PT-INR), estimated glomerular filtration rate (eGFR), intraoperative blood loss, and operation time.

### 2.4. Outcomes Evaluated

We assessed postoperative mortality and morbidity (greater than Clavien-Dindo Grade 3), PHLF, and postoperative hospital stay. PHLF was categorized according to the criteria of the International Study Group of Liver Surgery (ISGLF) [5]. Patients were also categorized as meeting or not meeting Makuuchi's criteria.

### 2.5. Statistical Analysis

Data are expressed as median and range. Differences between two groups were assessed by the Mann-Whitney *U* test and *χ* ^2^ test. Differences between three groups were assessed by one-way analysis of valiance and the Tukey multiple comparison procedure. Predictive value was assessed by calculating the area under the receiver operator characteristic (ROC) curve (AUC). Statistical analysis was performed using SPSS Statistics version 22.0 (IBM, NY, USA). A *P* value < 0.05 was considered statistically significant.

### 2.6. Study Design

The study design was approved by our institution's ethics review board (approval number 739) and the need for informed consent was waived in view of its retrospective nature.

## 3. Results

**Figure 1:**
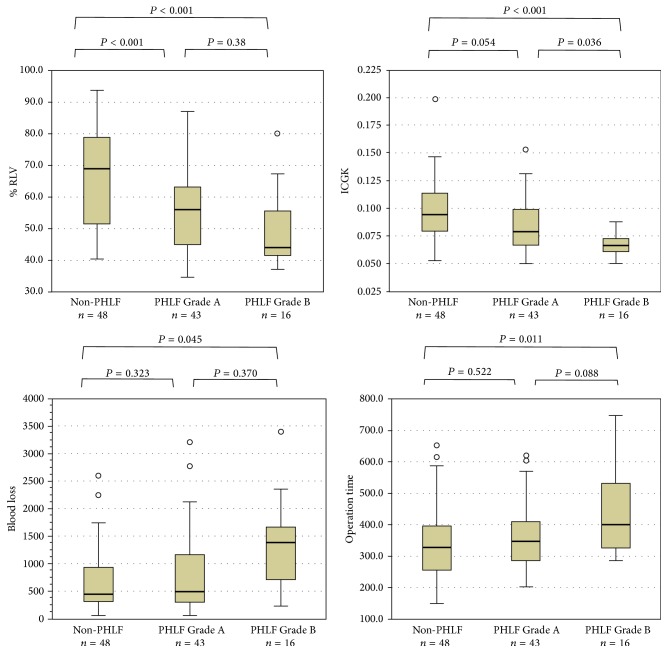
Distribution of factors significantly associated with development of PHLF. The rICGK of patients who developed PHLF Grade B was significantly lower than that of others. Blood loss volume is greater and operation time is longer in the PHLF Grade B than Grade A group, although not significant.

**Figure 2:**
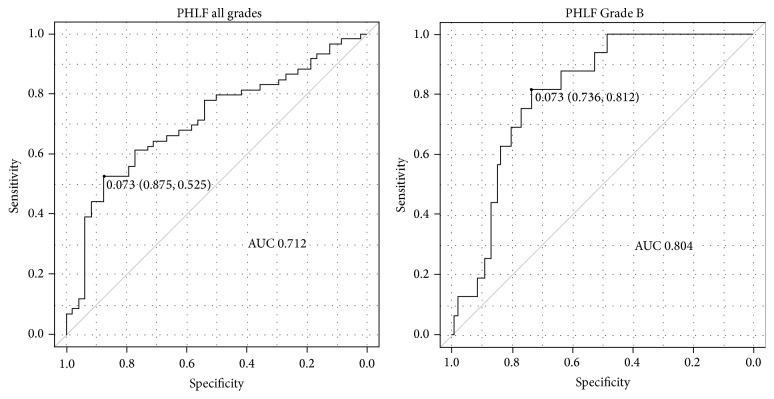
Receiver operating characteristic curve of rICGK for prediction of PHLF. Low rICGK has high predictive value for development of PHLF Grade B rather than PHLF of all grades (including PHLF Grade A).

**Figure 3:**
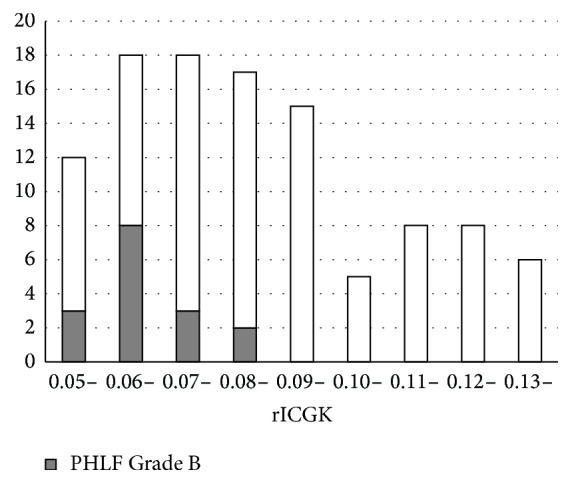
Histogram showing relationship between rICGK and PHLF Grade B. Patients whose rICGK was more than 0.09 did not develop severe PHLF (Grade B).

There was no postoperative mortality. There was one in-hospital death that was not directly related to hepatectomy (it was due to pleural dissemination of renal cell carcinoma). PHLF was identified in 59 patients (55.1%), being Grade A in 43 (40.2%), B in 16 (15.0%), and C in none. Patient characteristics according to PHLF Grade A or Grade B and absence of PHLF (non-PHLF) are shown in Table 3. There were significant differences between these three groups in %RLV, rICGK, operative blood loss, and operation time. A significant difference was also observed between PHLF Grades A and B for rICGK (Figure 1). A low rICGK was a good predictor of development of PHLF Grade B (AUC, 0.804; 95% confidence interval, 0.712–0.895) The optimal cut-off value of rICGK was 0.073 for predicting both PHLF Grade B (sensitivity, 0.812 specificity, 0.736) and PHLF all grades (sensitivity, 0.525 specificity, 0.875) (Figure 2): patients whose rICGK was greater than 0.09 did not develop PHLF Grade B (Figure 3).

Patients who developed PHLF Grade B had significantly longer postoperative hospital stays (14.5 versus 43.7 days, *P* < 0.001) and significantly higher rates of morbidity greater than Clavien-Dindo Grade 3 (8.3 versus 43.7%, *P* = 0.007) than those who did not develop PHLF. There were no significant differences in postoperative hospital days and rates of morbidity greater than Clavien-Dindo Grade 3 between PHLF Grade A and non-PHLF groups (16.5 versus 14.5 days, *P* = 0.429; 27.9 versus 8.3%, *P* = 0.062) (Table 4).

**Figure 4:**
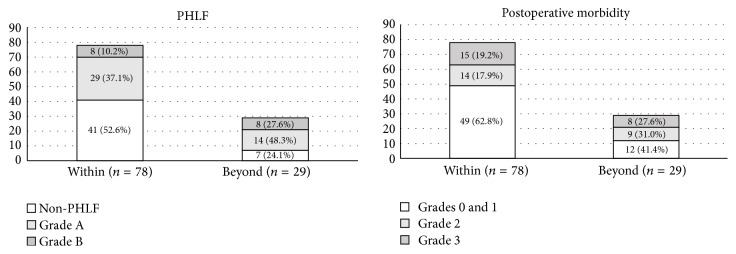
Postoperative morbidity according to Makuuchi's criterion. Patients who met Makuuchi's criterion had lower incidences of severe PHLF and postoperative morbidity than those who did not, although not significant.

The 29 patients who did not meet Makuuchi's criteria had significantly higher rates than those who did meet this criterion of developing PHLF (*P* = 0.016), but not of developing PHLF Grade B (*P* = 0.054) or morbidity greater than Clavien-Dindo Grade 3 (*P* = 0.350) (Figure 4).

## 4. Discussion

There was no postoperative mortality in this study. According to a Japanese national database, the perioperative mortality of hepatectomy performed for more than one segment (except for a lateral segment) is 4.0% [6]. Thus, the rICGK ≧ 0.05 criterion appears to be safe regarding zero mortality. However, there was a high incidence of PHLF. Previous studies have reported the incidence of PHLF as 9.0%–39.6% [7, 8]. Because patient characteristics have varied between studies, it is not valid to simply compare our findings with those of other studies; however, this is a noteworthy issue. Many of the cases of PHLF in the present study were PHLF Grade A, which had relatively little influence on the patients' postoperative course. However, patients who developed PHLF Grade B had a high morbidity rate and longer postoperative hospital stay. Because major operative blood loss and long operating time are considered to contribute to development of PHLF, surgeons should try to minimize blood loss and improve their surgical techniques. A low rICGK had good predictive value for development of PHLF Grade B in this study. The optimal cut-off value was 0.073 for predicting PHLF Grade B and patients with rICGK ≧ 0.09 did not have any severe PHLF in our series. Because rICGK was used not to predict PHLF but to assess the eligibility for hepatectomy in our series, we think these values are not directly meaningful, but these findings are consistent with our clinical experience; Thus, patients with higher rICGK may not be at risk of severe PHLF and, in patients with rICGK < 0.07, more careful perioperative management should be performed to avoid PHLF. There was also a high incidence of postoperative morbidity greater than Clavien-Dindo Grade 3, which may at least in part be attributable to our perioperative management policy. We rarely place prophylactic drains after hepatectomy and perform CT scans routinely on postoperative Day 7. We frequently perform percutaneous drainage when we suspect a fluid collection. This policy results in a relatively high frequency of postoperative percutaneous drainage and these cases are counted as postoperative morbidity Grade 3 even when they do not actually have an infection or biliary leak.

We consider the rICGK ≧ 0.05 criterion to be more expansive than Makuuchi's criteria because 29 study patients who did not meet Makuuchi's criteria did meet the rICGK ≧ 0.05 criterion. Although these patients tended to have poorer postoperative outcomes than patients who did meet Makuuchi's criteria, this difference was not significant. How far we can expand the indications for hepatectomy is controversial. Iguchi and colleagues reported results of hepatectomy for HCC based on the criterion of rICGK more than 0.03 [8]. In their study, patients whose rICGK was less than 0.05 had a significantly higher incidence and greater severity of PHLF than patients whose rICGK was more than 0.05. However, these two groups did not differ significantly in perioperative mortality or long-term oncological outcomes.

Patients with lower rICGK have a higher operative risk. However, because hepatectomy is the only potentially curative treatment modality for many liver malignancies, it is difficult to determine the optimal operative risk. The appropriate lower limit for rICGK may be different according to the underlying disease; we had too few patients in this study to assess this possibility.

In 2011, PHLF grading was proposed by the ISGLF. Since then, cross-sectional research on PHLF has become possible and such studies are increasingly being performed. Further accumulation of data and prospective studies investigating criteria for hepatectomy are expected.

This study has several limitations. It was a retrospective study and factors such as performance status and comorbidities were considered when assessing operative indications. Thus, some poor risk patients may have been excluded. The small sample size resulted in low statistical power for rare morbidities such as PHLF Grade C.

In conclusion, the rICGK ≧ 0.05 criterion is a sufficiently broad and safe criterion for selecting patients with various diseases for hepatectomy.

## Figures and Tables

**Table 1:** Patient characteristics.

Variables	(*n* = 107)
Age, years	69 (38–86)
Sex, male, %	67.3
HBs antigen+, %	12.1
HCV antibody+, %	14.0
ICGK	0.151 (0.069–0.264)
ICGR15, %	11.9 (1.9–35.4)
TLV, mL	1181 (735–2169)
% RLV	59.3 (34.7–93.7)
rICGK	0.088 (0.050–0.199)
T-Bil, mg/dL	0.7 (0.2–2.5)
Alb, g/dL	3.9 (1.8–5.2)
Plt, 10^4^/*μ*L	20.5 (8.3–72.0)
PT-INR	1.03 (0.93–1.93)
eGFR (mL/min)	76.0 (6.5–185.6)
Blood loss, mL	867 (50–7750)
Operation time, min	361 (151–748)

Indications for hepatectomy	(*n* = 107)

Hepatocellular carcinoma	52
Metastatic liver tumor	29
Cholangiocarcinoma	14
Others	12

HBs antigen+: hepatitis B virus surface antigen positive, HCV antibody+: hepatitis C virus antibody positive, ICGR15: indocyanine green retention rate at 15 minutes, TLV: total liver volume, % RLV: remnant liver volume/total liver volume (%), T-Bil: total bilirubin, Alb: albumin, Plt: platelet count, PT-INR: international normalized ratio of prothrombin time, and eGFR: estimated glomerular filtration rate.

**Table 2:** Type of hepatectomy.

Type of hepatectomy	Total 107
Trisectionectomy	2
Hemihepatectomy	55
Right hemihepatectomy	34
Left hemihepatectomy	21
Sectionectomy	44
Right anterior + left medial	4
Right posterior	21
Left medial	10
Left lateral	9
Segmentectomy	6

Both trisectionectomies were right trisectionectomies. Segmentectomy included S3 (two patients), S2, S5, S6, and S5 + 6 (one patient each).

**Table 3:** Patient characteristics according to PHLF grade.

Variables	Non-PHLF (*n* = 48)	PHLF Grade A (*n* = 43)	PHLF Grade B (*n* = 16)	*P* value
Age, years	69 (38–82)	68 (40–86)	69 (56–93)	0.59
Sex, male, %	75.0	55.8	75	0.12
HBs antigen+, %	14.5	11.6	6.3	0.68
HCV antibody+, %	10.4	11.6	31.3	0.10
ICGK	0.154 (0.069–0.212)	0.156 (0.091–0.264)	0.137 (0.085–0.211)	0.21
ICGR15, %	9.9 (4.2–35.4)	10.2 (1.9–25.6)	12.9 (4.2–27.8)	0.39
TLV, mL	1214 (786–2025)	1133 (735–2086)	1320 (829–2169)	0.39
% RLV	68.9 (40.5–93.7)	55.9 (34.7–87.0)	44.0 (37.2–80.2)	<0.001
rICGK	0.095 (0.053–0.199)	0.079 (0.050–0.153)	0.067 (0.050–0.088)	<0.001
T-Bil, mg/dL	0.5 (0.2–2.5)	0.7 (0.3–1.9)	0.7 (0.4–2.2)	0.74
Alb, g/dL	4.1 (1.8–5.2)	4.1 (2.6–4.7)	3.9 (3.0–4.7)	0.31
Plt, 10^4^/*μ*L	19.4 (8.3–72.0)	17.0 (9.7–41.5)	17.7 (8.6–45.5)	0.68
PT-INR	1.03 (0.93–1.93)	1.03 (0.94–1.35)	1.06 (0.94–1.31)	0.93
eGFR (mL/min)	75.8 (53.2–124.4)	74.1 (6.5–186.0)	69.3 (38.9–120.4)	0.54
Blood loss, mL	440 (50–2600)	500 (70–7750)	1385 (230–3400)	0.047
Operation time, min	328 (151–654)	348 (204–620)	401 (286–748)	0.02

*P* values between the three groups were assessed by one-way analysis of variance and the Tukey multiple comparison procedure.

**Table 4:** Postoperative outcomes according to PHLF grade.

	ALL (*n* = 107)	Non-PHLF (*n* = 48)	PHLF Grade A (*n* = 43)	PHLF Grade B (*n* = 16)
Postoperative hospital stay (days)	16 (9–186)	14.5 (9–70)	16 (10–123)	34.5 (16–186)
Postoperative morbidity (≧G3), %	21.5	8.3	27.9	43.7

Postoperative morbidity greater than Clavien-Dindo Grade 3 was evaluated.
